# Optimization and Analysis of a U-Shaped Linear Piezoelectric Ultrasonic Motor Using Longitudinal Transducers

**DOI:** 10.3390/s18030809

**Published:** 2018-03-07

**Authors:** Hongpeng Yu, Qiquan Quan, Xinqi Tian, He Li

**Affiliations:** School of Mechatronics Engineering, Harbin Institute of Technology, Harbin 150001, China; 17S008010@stu.hit.edu.cn (H.Y.); tianxinqi@hit.edu.cn (X.T.); 17B908020@stu.hit.edu.cn (H.L.)

**Keywords:** piezoelectric ultrasonic motor, finite element method, longitudinal vibration, high power density

## Abstract

A novel U-shaped piezoelectric ultrasonic motor that mainly focused on miniaturization and high power density was proposed, fabricated, and tested in this work. The longitudinal vibrations of the transducers were excited to form the elliptical movements on the driving feet. Finite element method (FEM) was used for design and analysis. The resonance frequencies of the selected vibration modes were tuned to be very close to each other with modal analysis and the movement trajectories of the driving feet were gained with transient simulation. The vibration modes and the mechanical output abilities were tested to evaluate the proposed motor further by a prototype. The maximum output speed was tested to be 416 mm/s, the maximum thrust force was 21 N, and the maximum output power was 5.453 W under frequency of 29.52 kHz and voltage of 100 V_rms_. The maximum output power density of the prototype reached 7.59 W/kg, which was even greater than a previous similar motor under the exciting voltage of 200 V_rms_. The proposed motor showed great potential for linear driving of large thrust force and high power density.

## 1. Introduction

Piezoelectric ultrasonic motors (PUMs) have developed rapidly in the recent 40 years with the increasing demands of the actuators in engineering applications [1,2]. They drive the runners or rotors with the frictional forces that are generated by the specific movements of the driving feet, which are excited via the vibrations of the piezoelectric elements. They show the merits of small size, simple structure, high power weight ratio, high accuracy and resolution, quick response, self-locking when power off, and a lack of electromagnetic radiation [3,4,5,6]. Hence, they have been successfully used in the field of high-tech and civilian use, such as robot driving, high precision machine, micro electro mechanical system (MEMS), and digital camera autofocus system [7,8].

The elliptical-trajectory movements of the driving tips are generally generated for the purpose to obtain the stable frictional forces to drive the runners or rotors [9]. PUMs can be divided into three types: traveling wave ultrasonic motors [10,11,12,13,14,15,16,17], standing wave ultrasonic motors [18,19,20], and composite vibrations ultrasonic motors [21,22], according to the ways to generate the elliptical-trajectory movements. Generally, the PUMs using composite vibrations have simple structures, large thrust force, and high output speed, so they have become more popular in recent years. Furthermore, this type of PUMs usually uses the modes of longitudinal and bending hybrid vibrations [23,24], longitudinal and torsional hybrid vibrations [25,26,27], two orthogonal longitudinal hybrid vibrations [28,29,30,31,32], and two orthogonal bending hybrid vibrations [33,34,35,36] to form the elliptical-trajectory movements.

Liu et al. proposed a very novel linear ultrasonic motor using the hybrid of two orthogonal longitudinal vibrations [37]. Their motor had three transducers, and two vibration modes which were orthogonal in space and had a phase shift of 90° in time were selected to generate the elliptical movements on the driving feet. The prototype had a total length of 170 mm, height of 107.5 mm, and weight of 1.42 kg. The typical mechanical output abilities of it were the no-load output speed of 854 mm/s and the maximum thrust force of 40 N with the voltage of 200 V_rms_. However, this ultrasonic motor was large in size and complicated in fabrication.

This work proposed a novel U-shaped PUM focusing on high power density, miniaturization, and simple assembly technique. The novel PUM also used a bolt-clamped structure to get the high output speed and large thrust force. However, a lot of changes had been designed for the purpose of optimization, which included smaller dimensions, lighter weight, simpler assembly processing, and higher output power density. In addition, the maximum output speed and the maximum output thrust force had very important influence on the characteristics of the PUM, so they were considered adequately, too. The structure and operating principle were discussed to describe the novel PUM. The simulated analyses, including modal analysis and transient simulation, were developed to tune the resonance frequencies and obtain the elliptical movements of the particles on the driving surfaces. The vibration mode shapes and mechanical output abilities were tested to evaluate the PUM further after fabricating a prototype. The comparison of the performances between the novel PUM and the previous similar one was given and discussed in the end to draw the conclusion of this work. 

## 2. Structural Optimum of the PUM

**Figure 1:**
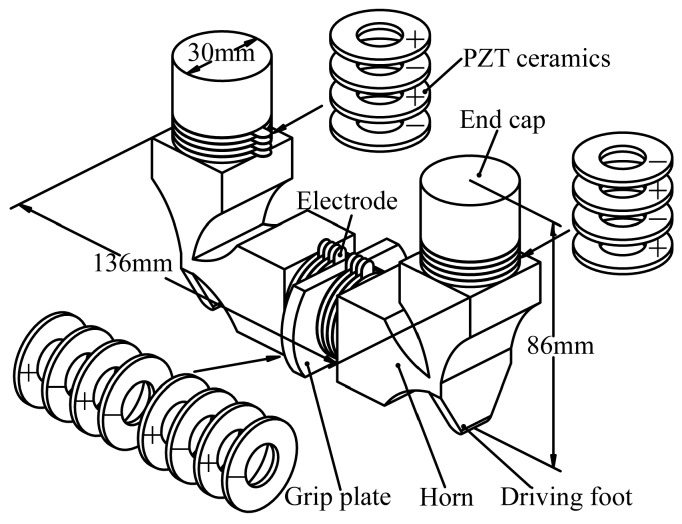
The three-dimensional structure of the modified piezoelectric ultrasonic motors (PUM).

The three-dimensional structure and overall dimensions of the proposed motor is shown in Figure 1. The PUM consists of one horizontal transducer and two vertical ones located in U-shape, which are designed to work with their longitudinal resonant vibration modes. The bolt-clamped structure is adopted in order to obtain the high output speed and large thrust force. Strong preload can be applied on the ceramics and fatigue of the adhesive layer can be avoided by using this structure. The longitudinal vibration modes of the transducers are excited by the d_33_ working mode of PZT ceramics, which is most direct and efficient because of its high electromechanical coupling efficiency. All the PZT ceramics are polarized along their thickness directions, and their polarizations are also shown in Figure 1. The horns of the PUM are designed into exponential shapes to magnify the vibration amplitudes and velocities, so that more mechanical vibration energies can enter the frictional coupling process. Besides all above, there are some beryllium bronze sheets serving as electrodes for applying the exciting voltage on the ceramics.

**Figure 2:**
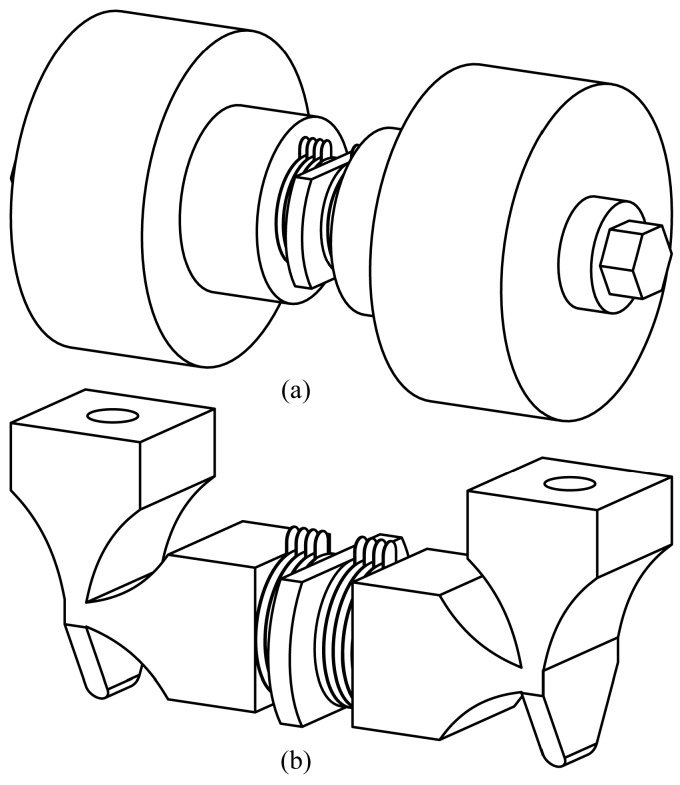
The assembly technique of the modified PUM: (**a**) The structure before secondary processing; and, (**b**) The structure after secondary processing.

The structures mentioned above are similar to the previous motor proposed by Liu et al. [37]. However, there are many obvious differences. First of all, the dimensions of the novel PUM are decreased in comparison with the previous one. As a result, the volume and weight of the motor can be reduced, which is to be the benefit of engineering applications. The decrease of dimensions will lead to the increase of resonance frequencies in the light of vibration theory, so the working frequency of the novel motor will get higher. It may be conducive to the improvement of the output speed and the output power density. Secondly, the assembly technique of the novel motor has been simplified a lot. It is known that the two horns were connected with a stud bolt in the previous structure that was designed by Liu et al. This structure causes the result that the pretightening moment of the bolt is difficult to control and the two driving feet cannot be assured to be parallel to each other, which will result in the poor performance of the PZT ceramics and the abrasion of the driving surfaces. So, the assembly technique of secondary processing is adopted in the novel structure to solve the problem. The motor should be fabricated as the structure showed in Figure 2a preliminarily, and it can then be machined into the structure showed in Figure 2b with the method of Wire Electric Discharge Machining (WEDM). In this way, the pretightening moment of the bolt can be controlled easily, and the depth of parallelism between the two driving feet can be up to a very high level, which is beneficial to simplify the assemblage and to improve the performances of the PUM. In addition, the end caps and the PZT ceramics are all designed into cylinder-shape, which makes the machining progress easier and the assembly precision higher. This change can reduce the cost drastically. Furthermore, the bumped plates on the flange are designed for the gripper to fix the motor and to apply preload force between the motor and runner. They could provide reliable and high-stiffness clamping, which is good for generating better vibration modes and performances. Last but not least, the driving feet are machined into trapezoid-shape to ensure the particles on the contact surfaces have close vibration amplitude. The tips of the driving feet are designed into cylinder-shape to ensure the contact area between the driving feet and runner remains unchanged during the vibration progress. All in all, these optimizations of structure are all designed to achieve the objective elaborated above.

## 3. Operating Principle of the Driving Effect

**Figure 3:**
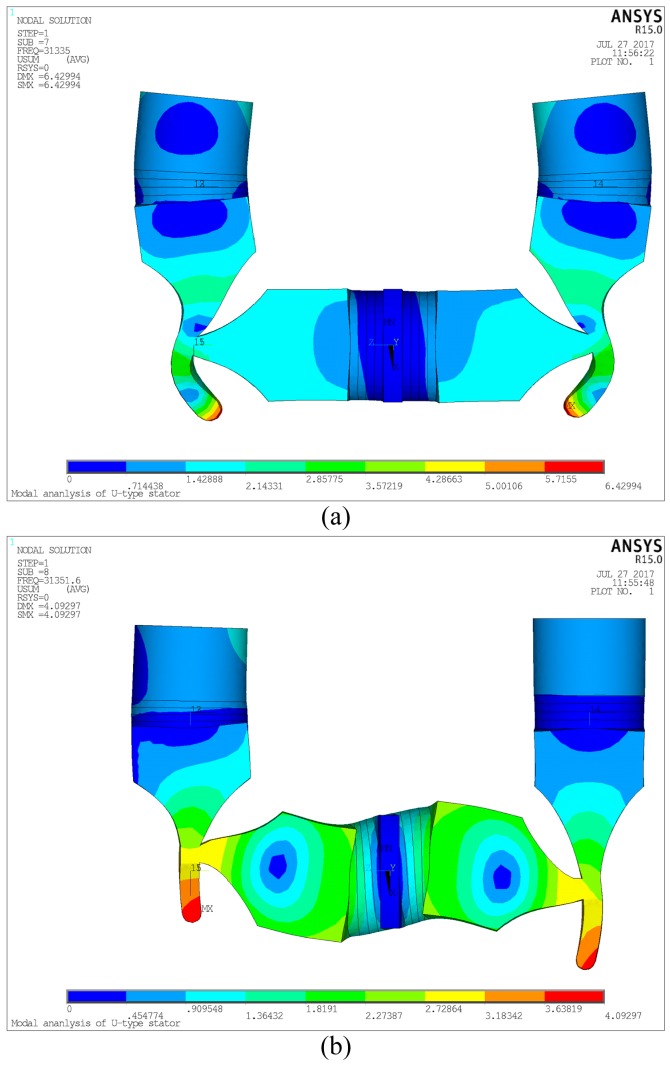
Vibration modes of the PUM: (**a**) Mode A; and, (**b**) Mode B.

The vibration modes must be selected reasonably in order to generate the elliptical movements with driving effect on the driving feet. According to the vibration theory, the higher the vibration order is, the smaller the vibration amplitude will be. So, the vibration order should be as low as possible to gain the high output speed and efficiency. At the same time, the vibration modes have to satisfy the essential requirements of PUM. Hence, the two vibration modes that are shown in Figure 3 are selected to superimpose the elliptical movements. Mode A in Figure 3 is the vibration mode that is excited by the longitudinal vibration of the horizontal transducer, which results in the horizontal displacements of the driving feet; mode B in Figure 3 is the vibration mode that is excited by the longitudinal vibrations of the vertical transducers, which results in the vertical displacements of the driving feet. The vibrations are both caused by the stretching-contracting movements of the PZT ceramics when alternating voltage is applied on them. As seen in Figure 3a,b, the vibration directions of the two driving feet are both converse, which are ensured by the structure of the PUM and the polarization of the PZT ceramics, respectively. Besides, the actual vibrations of the transducers are longitudinal-bending vibrations, because the coupling vibrations occur in the PUM. When the resonance frequencies of the two modes are nearly equivalent, the superposition of them will happen with the excitation of specific voltage.

**Figure 4:**
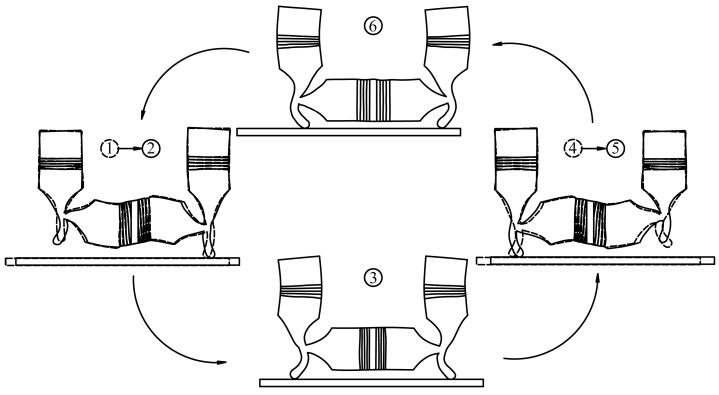
The working principle of the proposed PUM.

The resultant motion will be an ellipse if the harmonic vibrations of the particles on the driving feet are perpendicular in space and have a phase shift of π/2 in time basing on the knowledge of Lissajous figures. So that the movements that are required on the contact surfaces between the motor and runner with driving effect can be obtained. Furthermore, the motions of the two feet have a phase shift of π in time. Therefore, the two driving feet can contact with the runner and push it alternatively. The transducers are operated as the steps shown in Table 1 in one vibration period according to the selected vibration modes, and the working principle can be expressed as Figure 4 intuitively. The deformation of the motor changes from status 1 to status 2 when *t* is before and after *nT* according to Table 1 and Figure 4. The right driving foot keeps contacting with the runner and pushing it with frictional force in one direction in this process. The deformation of the motor becomes status 3 when *t* reaches (*n* + 1/4) *T*. The two driving feet both contact with the runner and the motor has no driving effect at this moment exactly. Then, the deformation of the motor changes from status 4 to status 5 when *t* is before and after (*n* + 1/2) *T*. The left driving foot keeps contacting with the runner and pushing it with frictional force in the direction that is identical with the right one in this process. The deformation of the motor becomes status 6 when *t* reaches (*n* + 3/4) *T*. The two driving feet both contact with the runner and the motor has no driving effect at this moment exactly. As a result, the elliptical-trajectories of the particles on the driving feet are generated in the circulation from status 1 to status 6, and the runner is pushed to move in a specific direction. Furthermore, the elliptical motions will be reversed, and the motion in the opposite direction of the runner can be realized on condition that the phase shift of the two vibration modes changes from π/2 to −π/2.

## 4. Dimensional Design and Simulation Analysis of the PUM

It is clear that the resonance frequencies of the two selected vibration modes must be nearly equivalent to ensure the proper operation with the aforementioned operating principle. So, the structural parameters should be designed elaborately to accomplish this objective. FEM was used to tune the resonance frequencies by modal analysis with ANSYS software. The finite element modal with the designed materials was conducted as the structure showed in Figure 1. The exciting voltage of the PZT ceramics was set as zero, and Block Lanczos method was used to extract the analysis results. The selected vibration modes were excited by applying the fixing boundary conditions on the surfaces of the flange. The optimal structural parameters were obtained after several times of iteration, and the resonance frequencies of the longitudinal vibrations of the horizontal and vertical transducers were calculated to be 31.335 kHz and 31.352 kHz, respectively. There was a very small discrepancy of 0.017 kHz between the two frequencies, so that the conditions of superimposing Lissajous Figures could be satisfied and the resonance frequency of the whole motor could be seen as 31.3 kHz, theoretically. However, the real resonance frequency and the optimal working frequency must be tested in the experiments.

**Figure 5:**
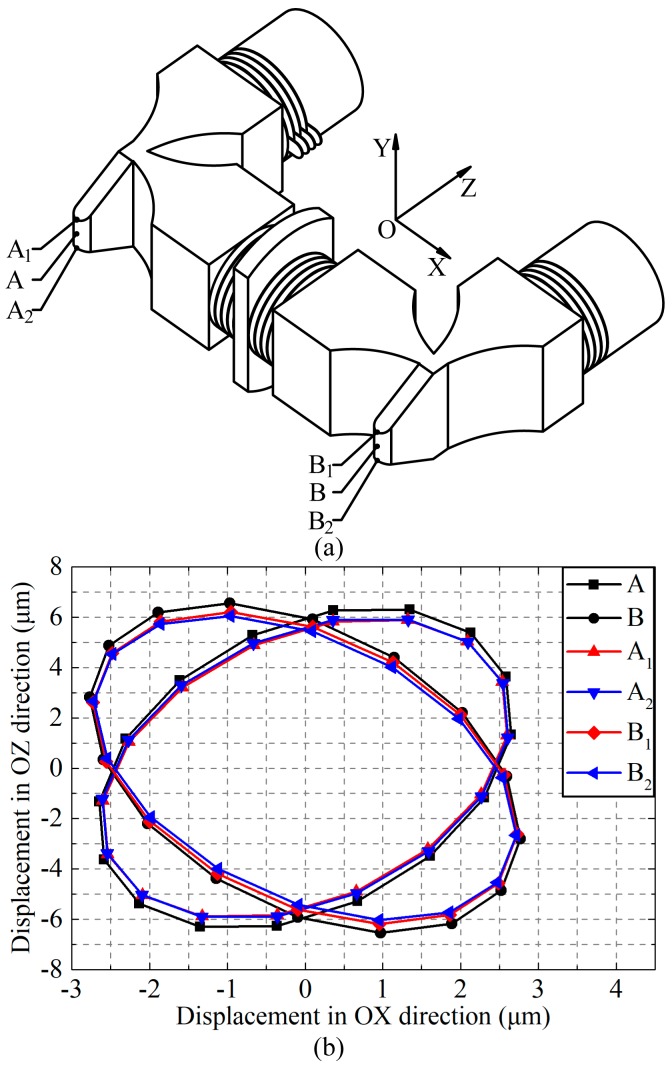
The motion trajectories of the driving surfaces: (**a**) Point selections; and, (**b**) Motion trajectories of the selected points.

The transient simulation was developed based on the structural parameters that were designed by modal analysis to investigate the motion behaviors of the particles on the driving surfaces. Sine and cosine exciting voltages with effective value of 100 V and frequency of 31.344 kHz were applied on the two groups of PZT ceramics in the simulation according to the operating principle illuminated above. The motion trajectories in the XOZ plane of the selected points on the two driving surfaces can be obtained when the status of the vibration got stable (after about 3 ms of simulation time). The point selections are shown in Figure 5a and the motion trajectories are shown in Figure 5b. The phenomenon can be recognized clearly as follows, according to Figure 5. Firstly, the motion trajectories of the points on the driving surfaces are ellipses exactly, which agrees with the theoretical analysis. As a result, it is preliminarily verified that the dimensional design is correct and the driving effect of the PUM is feasible. Secondly, the motion trajectories of different points on the driving surfaces are nearly concordant, which is a great improvement. This improvement can decrease the relative sliding between the PUM and the runner, so that the efficiency of the friction coupling progress and the output speed of the PUM will be increased. The improvement of the characteristic will be measured in the experiments. In addition, there is a phenomenon that is different from the theoretical analysis that the axes of the two ellipse-trajectories are not coincident with each other. It is because that the coupling bending vibrations between different transducers and the phase lags that are caused by structural damping exist in the PUM, and they are different in the three transducers. Hence, the ideal conditions for the superimposition of Lissajous Figures cannot be satisfied, which results in that phenomenon. In spite of this, the motion still has driving effect and the influence on the characteristic can be evaluated in the experiments.

## 5. Experimental Evaluation of the PUM

**Figure 6:**
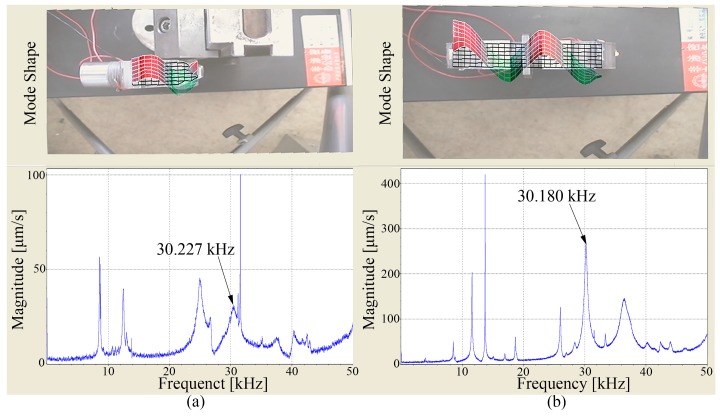
The results of vibration scanning: (**a**) The coupling bending vibrations of the vertical transducers; (**b**) The coupling bending vibration of the horizontal transducer.

A prototype was fabricated to conduct the experiment for the purpose of evaluating the characteristics and validating the improvement of the optimized PUM. The vibration characteristic was a crucial factor to impact the driving performance of the PUM, so an SLDV (Scanning Laser Doppler Vibrometer) was used to measure the vibration characteristic of the prototype. The prototype was fixed by the grip plate when the exciting voltage was applied on it, and the test region should be perpendicular to the laser. The coupling bending vibrations excited by the longitudinal ones were measured considering the simplification and accuracy of the measurement. The vibration mode shapes and vibration velocity response spectrums are shown in Figure 6.

Figure 6a shows the vibration characteristic of mode A. In view of the accuracy of the measurement, the plane part of the surface was selected as the test region. Figure 6b shows the vibration characteristic of mode B. It can be known that the vibrations of the transducers are longitudinal-bending vibrations exactly according to the mode shapes, and the bending component is caused by the longitudinal one. The vibration mode shapes are in good agreement with the theoretical analysis, which verify the validity of the design and analysis. The response frequencies of mode A and mode B are tested to be 30.227 kHz and 30.180 kHz on the basis of the response spectrums, respectively. There is a difference of 0.047 kHz between the two frequencies, which indicates the good degeneration of the two selected vibration modes. The two response frequencies can be thought as identical with the ones from modal analysis by taking the error from simulation and experiment in consideration, and the working frequency of the PUM can be thought as about 30.2 kHz.

**Figure 7:**
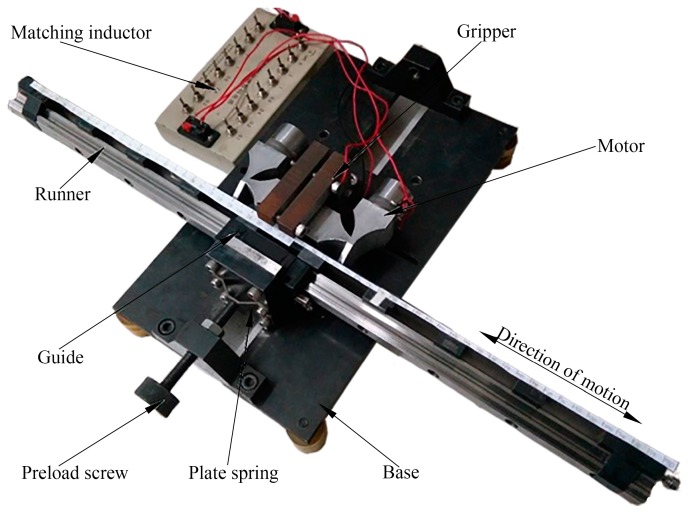
The prototype and experimental platform of the proposed PUM.

The mechanical output abilities of the prototype were tested with the instruction of the vibration test. An experimental platform was designed and fabricated for the prototype. The platform with the prototype is shown in Figure 7. The prototype was fixed on the platform by a gripper. A guide was adopted to ensure the linear motion of the runner. The preload between the driving feet and the runner was applied by a plate spring.

**Figure 8:**
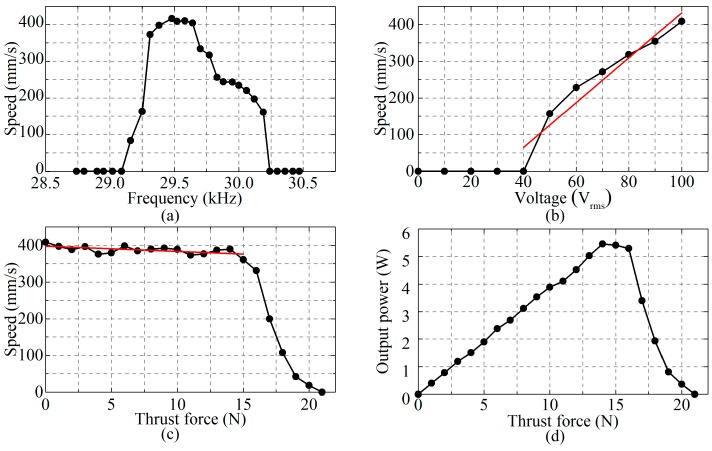
The mechanical output abilities of the prototype: (**a**) Frequency-speed characteristic; (**b**) Voltage-speed characteristic; (**c**) Thrust force-speed characteristic; and, (**d**) Thrust force-output power characteristic.

Firstly, the experimental installation was fabricated and the preload between the driving feet and runner was adjusted to be about 100 N to obtain the relatively best mechanical output ability, so as to achieve the maximum output power density. Then, the measurement of the frequency versus no-load speed was carried out with the following conditions: the phase shift of the exciting voltage was 90° and the amplitude of the exciting voltage was 100 V_rms_. The frequency-speed characteristic was obtained by changing the frequency of the exciting voltage from 28.74 kHz to 30.47 kHz, as shown in Figure 8a. The output speed is fastest when the frequency is about 29.4–29.7 kHz, according to the curve in Figure 8a, so the optimal working frequency of the prototype is set as 29.52 kHz, which has a very small difference from the modal analysis or vibration test. It is because of the errors that are caused by machining and simulation, but the influence of it is very small.

Next, the voltage-speed characteristic was measured in conditions of keeping the frequency of the exciting voltage being 29.52 kHz and the phase shift of the exciting voltage being 90°, as shown in Figure 8b. It can be found that the threshold voltage is about 40 V_rms_ and the no-load speed increases when the amplitude of the exciting voltage gets higher than the threshold voltage. Besides, the no-load speed and the amplitude of the exciting voltage fit a good linear relation, which makes the PUM easy to control in engineering applications.

Additionally, the thrust force was changed to measure the speed of the PUM with the frequency of the exciting voltage of 29.52 kHz, the amplitude of 100 V_rms_ and the phase shift of 90°. The thrust force-speed characteristic is shown in Figure 8c. It indicates that the physical characteristic of the PUM is very hard when the thrust force is smaller than 15 N, which is a good improvement of this PUM in engineering applications. The speed decreases when the thrust force is too big, and the fastest speed is no-load speed as 416 mm/s, the maximum thrust force is 21 N. Actually, the ideal characteristics of the PUM will be better than above when considering the friction between the guide and the runner.

The thrust force-output power characteristic of the prototype was obtained in accordance with the thrust force-speed characteristic, as shown in Figure 8d. The output power of the prototype increases first and then decreases with the increase of the thrust force. The maximum output power of the prototype can reach 5.453 W when the thrust force is 14 N. The weight of the prototype is 0.718 kg, so the maximum output power density of the prototype is 7.59 W/kg.

The mechanical output abilities of the prototype can be seen clearly according to the curves shown in Figure 8. Generally, a higher exciting voltage can be applied on the PZT ceramics and the preload between the driving feet and runner can be increased because the bolt-clamped structure can apply strong preload on the PZT ceramics and avoid the fatigue of the adhesive layer. As a result, faster speed and larger thrust force can be achieved, theoretically. Besides, applying the friction materials can also improve the performances of the PUM. These characteristics show the merits of this new configuration for linear driving and the good potential of the proposed PUM in engineering applications.

The comparison between the previous PUM [37] and the optimized one in this work is shown in Table 2. It can be known that the dimensions and weight of the PUM have been decreased and the quantity of the PZT ceramics has been reduced a lot, all of which accomplish the objective of miniaturization. The exciting voltage applied on the optimized PUM is 100 V_rms_, which is only half of the previous similar one; this condition makes the electric field densities of the PZT ceramics in these two motors be the same, under which these two motors can be evaluated with a good standard. This improvement reduces the demands of the power supply dramatically, which is a benefit to the integration of the whole driving system. The maximum speed of the optimized PUM is 49% and the maximum thrust force is 53% of the previous one with the voltage described above. This optimized PUM can also apply to the situations which need high speed and large thrust force because the output speed and the thrust force will increase with higher voltage and larger preload. At the same time, the output power will raise concomitantly with the increase of the exciting voltage. Hence, the objective of high power density is accomplished. The reasons causing the improvement can be summarized as follows basing on the modification of the PUM. First of all, the decrease of dimensions makes the resonance frequency of the optimized PUM get higher, which increases the vibration energy of the PUM with the same amplitude of voltage. Secondly, the motion trajectories of the particles on the driving surfaces have better conformance, so almost all of the particles on the interface can drive the runner synchronously, which increases the efficiency of the energy transforming in the friction coupling progress. In addition, the stiffness of the clamping condition in the new structure has been increased, so less vibration energy loses via the grip and larger preload can be applied on the driving surfaces. These factors above improve the performance of the PUM synthetically.

## 6. Conclusions

An improved and miniaturized U-shaped linear piezoelectric ultrasonic motor was proposed, fabricated, and tested, and the operating principle of the proposed PUM was analyzed. The new structure of the PUM was more suitable for machining and assembly, and the clamping condition got better than a previous motor. The dimensions were decreased and the weight was only about 50% of the previous one. The two selected vibration modes were analyzed and the resonance frequencies of them were tuned to be very close to each other by adjusting the structural parameters with modal analysis. The elliptical motion trajectories of the driving feet were obtained by transient simulation to verify the feasibility of the driving effect. The results of vibration test were in good agreement with the modal analysis, and the resonance frequencies of the two vibration modes were tested to be 30.227 kHz and 30.180 kHz, with a small discrepancy of 0.047 kHz. The typical output abilities of the prototype showed that the optimal working frequency was about 29.52 kHz with voltage of 100 V_rms_. The maximum output speed and maximum thrust force were tested to be about 416 mm/s and 21 N, respectively. The maximum output power was about 5.453 W and the power density was about 7.59 W/kg, which meant that the power density of the optimized PUM could reach the same level with only half amplitude of the exciting voltage being applied on the previous similar one. All of the results indicate that the desired optimization has been accomplished. Besides, a novel way to improve the power density of the PUM can be summarized from the design procedure in this work.

## Figures and Tables

**Table 1:** The vibration statuses of the transducers in one period.

Time	Left Vertical Transducer	Right Vertical Transducer	Horizontal Transducer	Vertices on Left Feet	Vertices on Right Feet
*t* = *nT* ^1^	Shortened	Extended	Normal	Top	Bottom
*t* = (*n* + 1/4) *T*	Normal	Normal	Shortened	Left	Right
*t* = (*n* + 1/2) *T*	Extended	Shortened	Normal	Bottom	Top
*t* = (*n* + 3/4) *T*	Normal	Normal	Extended	Right	Left

^1^ *T* is the period, *t* is the time, *n* = 0, 1, 2…

**Table 2:** The comparison between the previous similar PUM and the optimized one.

Item	Previous PUM [37]	Optimized PUM
Working frequency (kHz)	23.59	29.52
Total length (mm)	170	136
Total height (mm)	107.5	86
Weight (kg)	1.42	0.718
Quantity of PZT ceramics (mm^3^)	51411	17693
Amplitude of exciting voltage (V_rms_)	200	100
Maximum speed (mm/s)	854	416
Maximum thrust force (N)	40	21
Maximum power (W)	10.39	5.453
Maximum power density (W/kg)	7.32	7.59
